# Synthesis and Physical–Chemical Characterization of a Biopolymer Derived from Cassava Starch, Cashew Nutshell Liquid, and Diammonium Phosphate

**DOI:** 10.3390/polym17091184

**Published:** 2025-04-26

**Authors:** Manuel Acosta Humánez, Yair Vega Vega, Alvaro Arrieta Almario, Oriana Palma Calabokis, Jair de Jesús Arrieta Baldovino

**Affiliations:** 1 Research Group on Development and Innovation in Advanced Materials, University of Sucre, Sincelejo 700001, Colombia; manuel.acosta@unisucrevirtual.edu.co (M.A.H.); yair.vega@unisucrevirtual.edu.co (Y.V.V.); 2 Faculty of Engineering and Basic Sciences, Fundación Universitaria Los Libertadores, Bogotá 111221, Colombia; opalmac@libertadores.edu.co; 3 Civil Engineering Program, Universidad de Cartagena, Cartagena de Indias 130015, Colombia; jarrietab2@unicartagena.edu.co

**Keywords:** biopolymer, CNSL, DSC/TGA, starch, XRD, UV measurements

## Abstract

A biopolymer was synthesized using starch, cashew nutshell liquid (CNSL), and the commercial fertilizer diammonium phosphate (DAP). The biopolymer and its constituents were characterized using SEM, infrared spectroscopy, X-ray diffraction (XRD), ultraviolet–visible diffuse reflectance spectroscopy (UV-Vis DRS), and thermal analysis by TGA and DSC. The results showed that fertilizer particles could be encapsulated by the starch and CNSL matrix. Functional groups and ions in the biopolymer showed characteristic bands associated with starch, CNSL, and DAP fertilizer. Moreover, the biopolymer diffraction peaks contained XRD peaks of starch and DAP. The crystallinity of the biopolymer decreased. Starch, CNSL, and DAP electronic transitions appeared in the biopolymer, with possible signal overlapping. The bandgap of starch and biopolymer did not differ significantly (6.19 and 6.16 eV, respectively). Both materials acted as insulators. Differential scanning calorimetry/thermogravimetric evidenced the materials’ thermal behavior, where water elimination, degradation, oxidation, and gas formation were registered.

## 1. Introduction

The use of fertilizers in agriculture is crucial for crop growth and productivity. However, the rapid release of nutrients compared to the plant’s absorption rate can lead to environmental pollution, which implies the need for caution in fertilizer use [1]. The development of plants relies on primary nutrients such as nitrogen, phosphorus, and potassium, as well as micronutrients like calcium (Ca), iron (Fe), zinc (Zn), and copper (Cu) [2].

One of the world’s most important sources of phosphorus fertilizers is diammonium phosphate (DAP), a chemical compound with the molecular formula (NH_4_)_2_HPO_4_. It is produced by the reaction between ammonia and phosphoric acid [3]. The application of DAP in crops is convenient because it provides active and low-hygroscopic ingredients that help in plant metabolic processes, such as respiration and photosynthesis [3,4].

Nowadays, industries dedicated to the production and marketing of fertilizers face two interesting problems daily: the struggle to develop their products to increase the effectiveness of their application and to mitigate the adverse ecological effects, such as the emission of greenhouse gases, soil acidification, and eutrophication due to over-fertilization, that is, increasing the amount of fertilizer required to improve the absorption of the fertilizer in plants. This fact generates economic and resource losses [5,6].

As a solution to the losses caused by over-fertilization, using controlled-release or slow-release fertilizers (CRFs/SRFs) emerges as a promising strategy. These fertilizers slow down the bioavailability of nutrients, aligning it with the plant growth process. This not only reduces root damage compared to the conventional way but also optimizes the efficiency of the use of fertilizer. This promising approach offers a ray of hope for the future of sustainable agriculture [1,4,7,8,9].

Diverses CRF/SRF methodologies exist, including load carrier, physical or chemical reactions, and encapsulation techniques [6]. In recent years, biopolymers have been widely used in fertilizer encapsulation processes, as they exhibit excellent potential due to their availability, degradability, and eco-friendly properties [1,8,10,11,12,13]. One of the biopolymers is starch, which has the highest abundance percentage. It is found in diverse plant sources such as cassava, yam, rice, corn, wheat, potato, and cereals [14,15].

**Figure 1:**
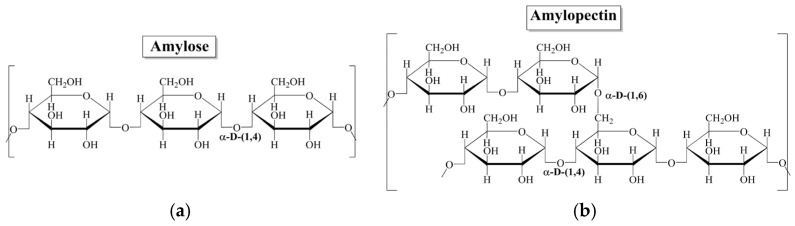
Starch chemical structure: (**a**) amylose, (**b**) amylopectin.

Structurally, starch is composed of two types of glucans: amylose (20–25%) and amylopectin (70–75%), as can be seen in Figure 1 [13,16,17]. As shown in Figure 1a, the structure of amylose is a long linear chain of D-glucose formed by α-D-(1→4) glycosidic bonds associated with the crystalline region of starch. Amylopectin (Figure 1b) has a branched structure with α-D-(1→4) and α-D-(1→6) glycosidic bonds of D-glucose. This corresponds to the amorphous region of starch [10,15,18,19,20]. On the other hand, it is relevant to mention that the relative proportion of amylose and amylopectin in starch depends on the vegetable source used for its extraction [16].

The hydrophilic nature of starch makes it a susceptible material, as it has a high permeability to water vapor. To overcome this problem, it is convenient to incorporate a reinforcing material into the polymeric matrix [13]. For this reason, combining starch with cashew nutshell liquid (CNSL) is an attractive option that, as last mentioned, could significantly improve the physical and chemical properties of the biopolymer.

**Figure 2:**
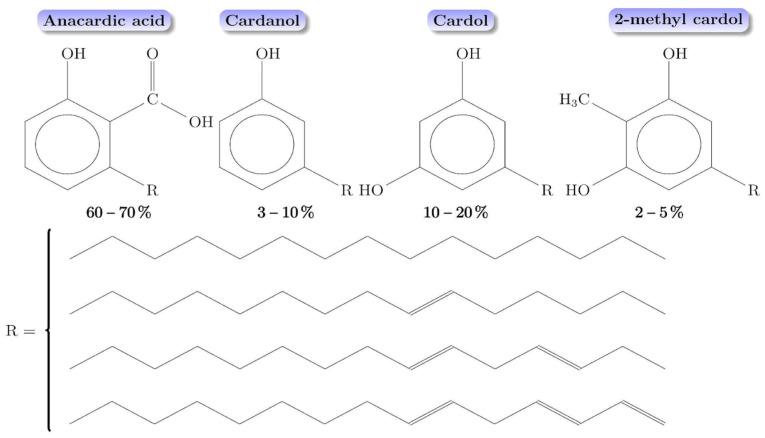
CNSL chemical compounds and their relative abundances. Data from Lomonaco and coworkers [26].

CNSL is obtained from the mesocarp shells of cashew nuts (*Anacardium occidentale* L.). This alkylphenolic mixture has a high viscosity and a reddish-brown color [21,22,23]. Cashew shell liquid represents about one-third of the mass of the nut. However, CNSL is relatively easy to obtain using some methods, such as mechanical extraction, solvent extraction, supercritical fluids, and vacuum distillation [24,25]. The chemical composition of CNSL is affected when heat is used; CNSL could be divided into natural and technical. Natural CNSL is extracted without heating and its composition is anacardic acid, cardanol, cardol, and traces of 2-methyl cardol, as shown in Figure 2. Technical CNSL is obtained by heating natural CNSL, and its composition is based on cardanol, cardol, and traces of 2-methyl cardol, since anacardic acid is decarboxylated, forming cardanol [25]. In general, CNSL-derived compounds, at a structural level, consist of salicylic acid (for anacardic acid), phenol (for cardanol), and resorcinol (for cardol), substituted with an aliphatic carbon chain (R) of 15 carbon atoms; these can be saturated or unsaturated, as shown in Figure 2. The presence of the methyl group in the last compound makes the difference with cardol.

Due to all the above, the purpose of this research was to encapsulate the diammonium phosphate fertilizer with the cassava starch-based biopolymer (*Manihot esculenta Crantz*) and cashew nutshell oil to study the interactions of the mentioned components and to understand their possible applications in agriculture as advanced slow-release fertilizer material. It is worth hightlighting there are no studies about SRF using a combination of starch and CNSL as a coating matrix and its application in agriculture as SRF. Therefore, this research represents the first attempt to incorporate CNSL into the coating material.

## 2. Materials and Methods

This work extracted starch from cassava (*Manihot Esculenta Crantz*), and the CNSL was obtained from the cashew nutshell. These extractions will be discussed subsequently. It is relevant to mention that the cashew nutshells were collected in the Pisa Bonito, Chinú (Córdoba), Colombia. In this town, there is an agroindustry dedicated to the production, processing, and marketing of cashew nut products called the Association of Cashew Producers of the Sabana (ASOPROMARSAB). Diammonium phosphate (DAP) was obtained commercially. Sodium hydroxide adjusted the water pH (99%, Sigma Aldrich, St. Louis, MO, USA). The pH measurements were carried out using a Mi 150 pH/temperature meter (Milwaukee Instruments, Inc., Rocky Mount, NC, USA) with a precision of ± 0.01 in pH value. Finally, the solution preparation and biopolymer synthesis were carried out using milli Q-grade ultrapure water.

### 2.1. Cassava Starch, Cashew Nutshell Oil Extractions, and DAP Fertilizer

**Figure 3:**
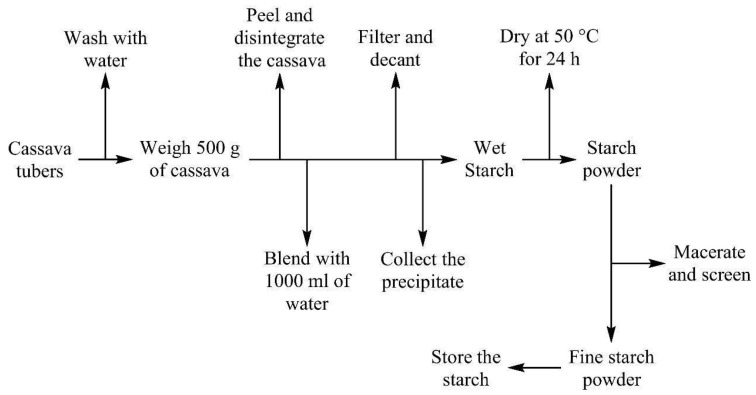
Flow chart of cassava starch extraction process.

In this study, starch from cassava was extracted using the traditional method, which is summarized in Figure 3. The cassava was washed, and 500 g was weighed, peeled, chopped, and blended with 1000 mL of distilled water. The obtained material was filtered with a muslin cloth, washed with 200 mL of distilled H_2_O three times, and decanted for 24 h. The precipitate was collected and dried at 50 °C. The cassava starch powders were sieved and macerated (60 μm mesh sieve), and the white powder obtained was stored for further analysis [27]. It is worth highlighting that a yield of approximately 20% by mass was obtained from this procedure. Figure 3 shows a flow chart of the cassava starch extraction process.

**Figure 4:**
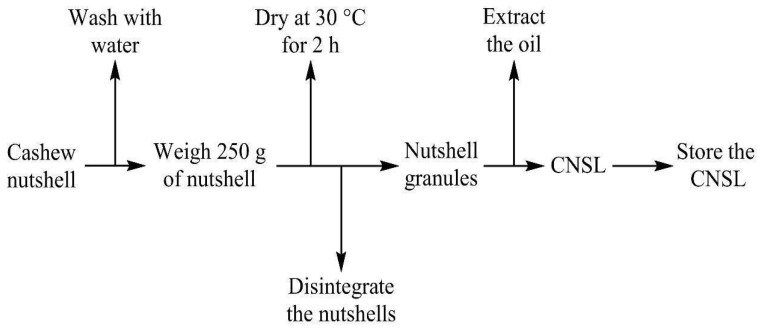
Flow chart of the CNSL extraction process.

CNSL was extracted from cashew nutshell by mechanical extraction, and the shells collected were washed with water. Then, 250 g of the nutshell was weighed and dried at 30 °C for 2 h. Afterward, the dry shells were chopped using a JTC Omniblender TM767 industrial blender (JTC ELECTRONICS CORP, Guangdong, China). Then, the chopped material was introduced in a hot–cold oil industrial steel U.S. SOLID oil press machine (US SOLID LLC, Plano, TX, USA) with a motor power of 350 W to obtain the substance of interest: oil [28]. It is worth highlighting that a yield of approximately 34% by mass was obtained from this extraction process. Figure 4 shows a flow chart of the CNSL extraction process.

### 2.2. Starch–CNSL–DAP Biopolymer Synthesis

**Figure 5:**
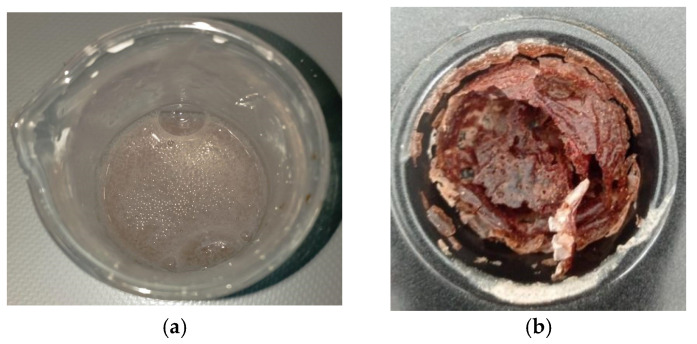
(**a**) Reaction mixture between cassava starch, cashew nutshell oil, and diammonium phosphate. (**b**) Biopolymer after 48 h heating.

**Figure 6:**
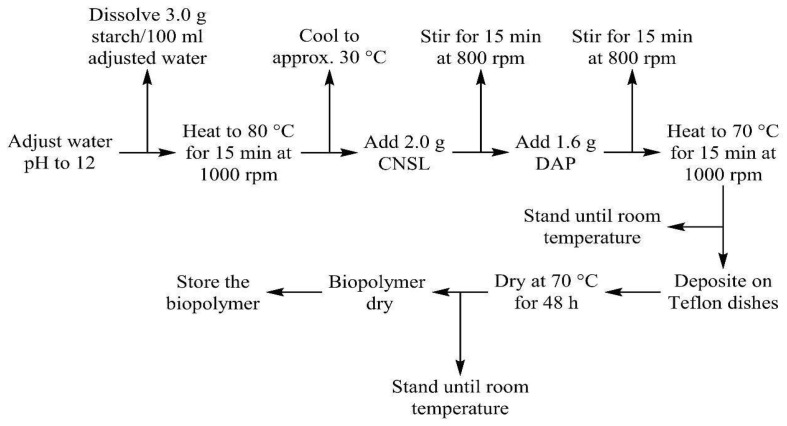
Flow chart of the synthesis of the starch–CNSL–DAP biopolymer.

The biopolymer synthesis developed as follows: the pH of ultrapure water was balanced to 12.0, using an aqueous solution of sodium hydroxide 0.1 M. Then, 3.0 g of cassava starch was dissolved in 100 mL H_2_O pH = 12.0. Afterward, it was heated to 80 °C with a stirring of 1000 rpm for 15 min. The above reaction mixture was left to stand at room temperature; 2.0 g of CNSL and 1.6 g of DAP were added and heated to 70 °C with stirring at 1000 rpm for 15 min (Figure 5a). The reaction mixture was placed in a polytetrafluoroethylene container and dried at 70 °C for 48 h. Finally, the biopolymer was removed with adequate precautions and stored [27]. Figure 5b shows the biopolymer obtained. Figure 6 shows the starch–CNSL–DAP biopolymer flow chart.

### 2.3. Characterization Techniques

Scanning electron microscopy (SEM) micrographs were obtained using a JEOL 5610 microscope (JEOL Ltd., Tokyo, Japan). The samples were treated with a 10 nm Au–Pd coating. A 0.10 nA current was applied for all the samples. The high acceleration voltage (HV) applied to the samples was 5.00 kV. An Everhart–Thornley detector (ETD) (JEOL Ltd., Tokyo, Japan) was used for all the measurements.

Chemical analyses were performed using attenuated total reflectance infrared spectroscopy (ATR-FTIR) using a Perkin Elmer Two spectrometer (PerkinElmer Inc., Shelton, CT, USA) in the 4000–400 cm^−1^ measurement range. The spectral resolution was 4 cm^−1^, and 24 scans were performed. The spectra of samples were recorded, normalized, and corrected through background (air).

X-ray diffraction measurements were registered with a Panalytical X’Pert PRO diffractometer (Malvern Panalytical Ltd., Almelo, The Netherlands) with KαCu radiation (λ = 1.540598 Å) and Bragg–Brentano geometry. This instrument ran continuously in a °2θ position goniometer from 5° to 90°.

Ultraviolet–visible (UV) diffuse reflectance spectroscopy (DRS) measurements were performed using a Varian Cary 5000 UV-Vis spectrometer (Agilent Inc., Santa Clara, CA, USA). The wavelength scanning interval was 2500 nm to 200 nm. A measurement time per step of 0.1 s and a step interval time of 0.1 nm were used. All the samples measured were solids.

Differential scanning calorimetry (DSC) and thermogravimetric analysis (TGA) were conducted using a Perkin Elmer STD6000 simultaneous thermal analyzer (PerkinElmer Inc., Shelton, CT, USA). The sample analyses were carried out in alumina crucibles under a nitrogen atmosphere with a 10 mL/min flow rate and a 10 °C/min heating rate ranging from 35 to 1000 °C. The mass of the analyzed samples was 5.735 mg for the starch, 16.401 mg for the CNSL, 7.951 mg for the DAP, and 12.823 mg for the CNSL–DAP–starch-based biopolymer. Furthermore, 30 min before measurement, a nitrogen purge was started to establish an inert environment to prevent any unnecessary oxidative decomposition.

## 3. Results and Discussion

### 3.1. Scanning Electron Microscopy Analysis

**Figure 7:**
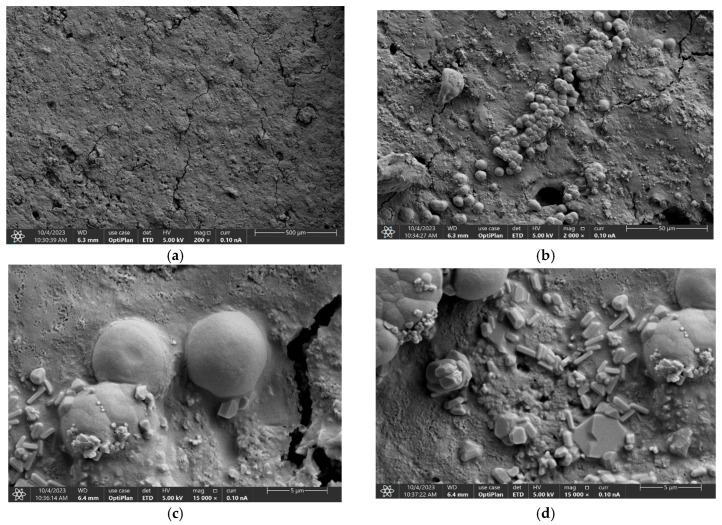
SEM micrographs for the starch–CNSL–DAP biopolymer measured at different depths: (**a**) 500 µm, (**b**) 50 µm, (**c**) 5 µm, and (**d**) 5 µm from another sample region.

Figure 7 shows SEM micrographs for the starch–CNSL–DAP biopolymer, measured from 500 µm to 5 µm. Surface cracks were observed in the synthesized biopolymeric material (Figure 7a), formed after drying. These micrographs are crucial in understanding the surface characteristics and the distribution of DAP fertilizer particles in the synthesized biopolymeric material. Surface cracks were observed in the synthesized biopolymeric material (Figure 7a), formed after drying. At 50 µm, as shown in Figure 7b, the presence of DAP fertilizer is evidenced by an inhomogeneous particle size distribution. Figure 7c,d are SEM micrographs measured at the same depth (5 µm) but from different sample regions. The micrograph shows DAP fertilizer particles embedded in the biopolymer. Table 1 exhibits the semiquantitative chemical analysis measurements of the starch–CNSL–DAP biopolymer. The results indicate that the sample’s most abundant chemical element percentages were carbon (C) and oxygen (O), which came from the polymeric matrix. Fluorine (F), sodium (Na), and phosphorus (P) could be associated with DAP fertilizer chemical compounds.

### 3.2. FTIR Analysis

**Figure 8:**
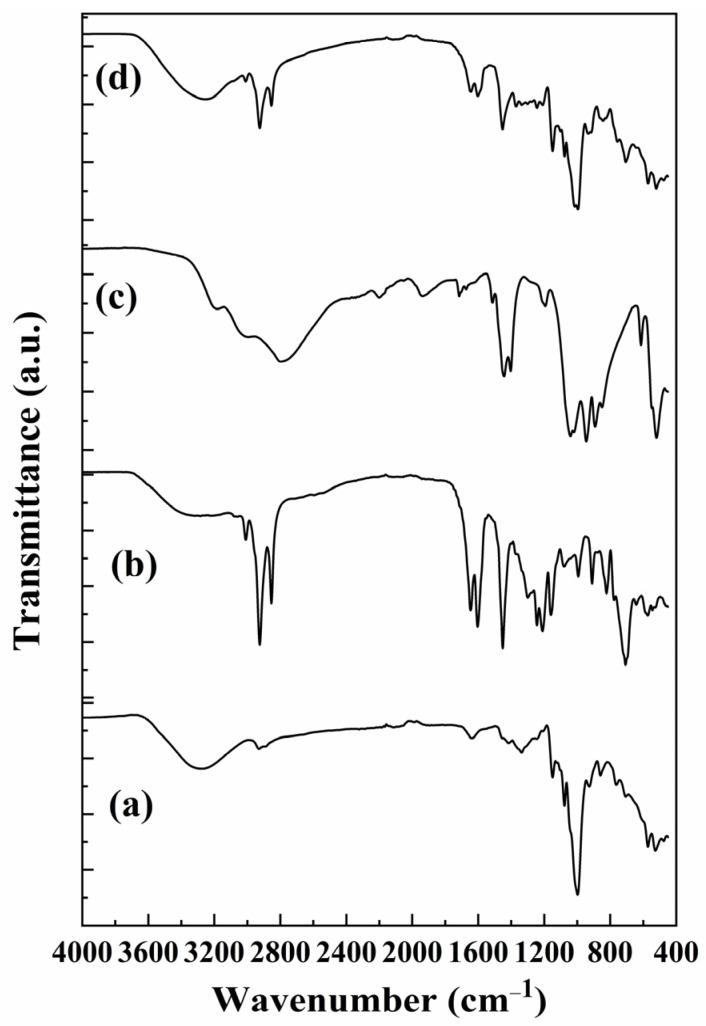
FTIR spectra for (**a**) starch, (**b**) CNSL, (**c**) DAP, and (**d**) starch–CNSL–DAP biopolymer.

The measured FTIR spectra for all the materials are shown in Figure 8. The starch spectrum (Figure 8a) indicates the presence of bands at around 3600–3200, 3000–2800, 1700–1600, 1200–1100, and 1100–900 cm^−1^. The related bands are associated with O-H, C-H, C=O, C-O-C, and C-O bonds, respectively. Moreover, the C-O-C bond in starch had a signal located at 900–700 cm^−1^ [29,30].

Several bands are shown in the CNSL spectrum (Figure 8b), and some bands overlap due to CNSL being a mixture of organic compounds. Well-defined bands are found at 3600–3200 (broad), 3050–3000, and 3000–2800 cm^−1^. These bands correspond to the O-H, stretching =C-H, and stretching C-H bonds, respectively. The latter signal is overlapped with the C-H alkane bond. Signals at 1600–1500 and 1500–1400 were associated with C=C and C=C-H bonding. These IR signals could overlap with C-O, C=O, and C-O-C bonds [31]. Figure 8c shows the FTIR spectrum of commercial fertilizer DAP. IR bands at approximately 2200 and 1936 cm^−1^ were associated with NH_4_^+^ ions [32]. Bands at 1700–1600 and 1600–1500 cm^−1^ are attributed to N-H bending and O-H bending vibrational modes. Signals at 1450–1350 cm^−1^ are associated with P=O stretching mode, and signals at 1070–920 cm^−1^ are attributed to P_4_O_6_ cage vibrational mode. This signal overlaps with the N-H symmetric deformation mode. Signals at 800–500 cm^−1^ are associated with P_4_O_6_ cage modes, while IR bands at 3400–2800 cm^−1^ are the sum of O-H and N-H bonds [33,34,35]. Figure 8d shows the FTIR spectrum of the starch–CNSL–DAP fertilizer, where the observed signals correspond to the sum of the bands obtained from the individual components, with the starch bands being the most defined and attributed to the functional groups in this chemical compound.

### 3.3. X-Ray Diffraction

**Figure 9:**
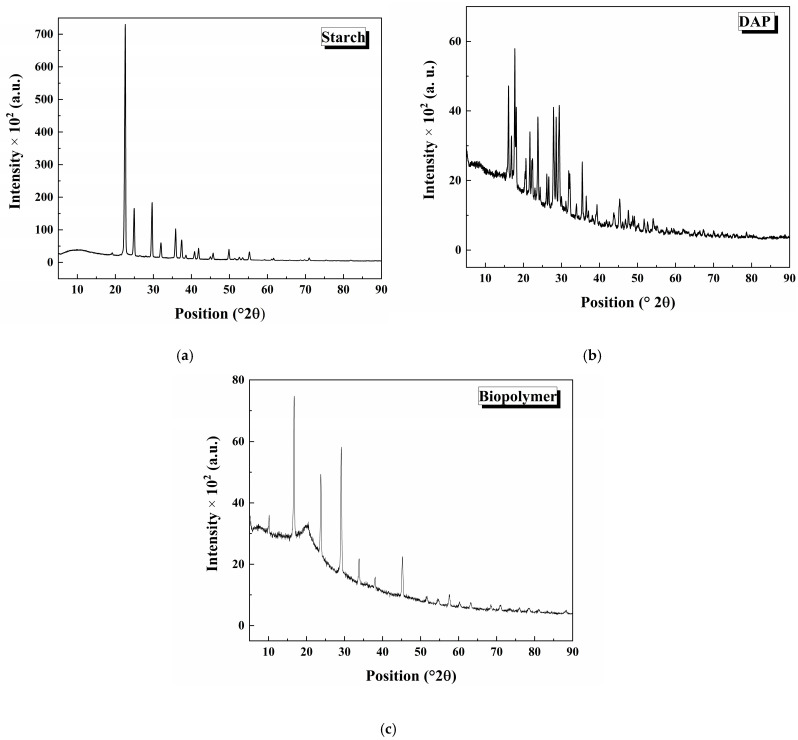
X-ray diffractograms for (**a**) starch, (**b**) DAP, and (**c**) starch–CNSL–DAP biopolymer.

The X-ray diffractogram for the individual components and the synthesized biopolymer is shown in Figure 9. Figure 9a shows the starch diffractogram measured. Two regions are observed: amorphous and crystalline. The amorphous region is attributed to amylopectin, while the crystalline region is associated with amylose. Generally, starch crystalline A and B types are directly related to amylose [36,37]. Diffraction peaks at 19.1, 22.6, 25.1, 29.6, 32.0, 36.0, and 37.5 °2θ are consistent with previous work, evidencing the starch’s A- and B-type crystalline amylose [36]. Then, it was possible to calculate the percentage of phases in starch according to the literature [38,39]. In this methodology, the crystalline percentage is defined as % crystallinity = (A_c_/A_t_)·100%, where A_c_ and A_t_ are the areas under the curve of crystalline and total XRD peaks. The amorphous region was 21.96%, and the crystalline region was 78.04%. The diffraction peak quantity in each phase evidences this behavior. Therefore, the XRD results in extracted starch show more amylose than amylopectin. Figure 9b shows the XRD diffractogram of DAP commercial fertilizer. The diffraction peaks are attributed to diammonium phosphate monoclinic and tetragonal phases or other impurities [40]. The starch–CNSL–DAP biopolymer diffractogram is shown in Figure 9c. It is observed that the intensity of the starch diffraction peaks decreases, and then the crystallinity of the sample diminishes. More intense DAP diffraction peaks are registered—some starch and DAP diffraction peaks overlap.

### 3.4. UV-Vis DRS Spectroscopy

**Figure 10:**
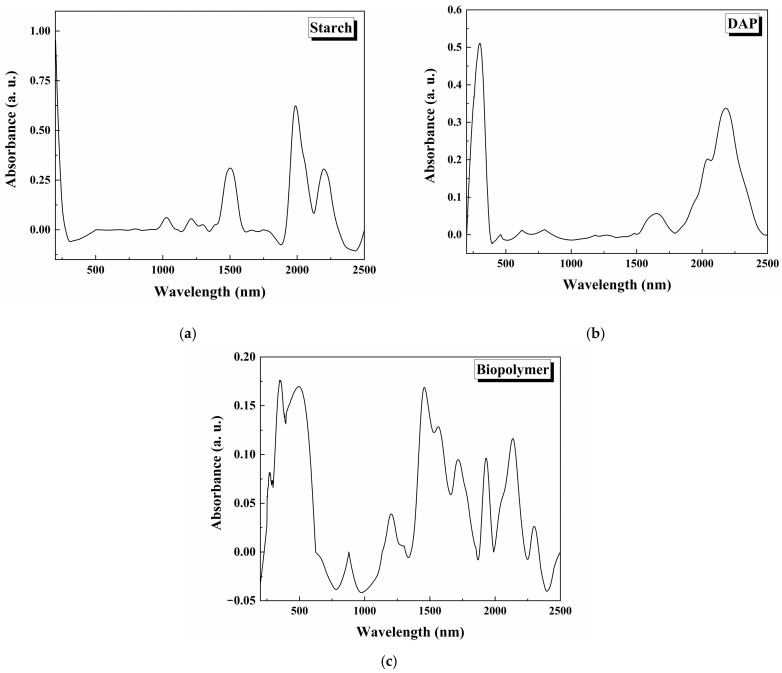
UV-Vis spectra in absorbance mode for (**a**) starch, (**b**) DAP, and (**c**) starch–CNSL–DAP biopolymer.

The UV-Vis spectra in absorbance mode for the starch, DAP, and starch–CNSL–DAP biopolymer are shown in Figure 10. The absorbance data were computed using the following expression: A = −log R, where R is the diffuse reflectance data of the samples [41,42]. The starch spectrum (Figure 10a) shows signals possibly associated with electronic transitions from functional groups in starch, such as σ → π*, π → π*, n → σ*, and n → π* [31]. Figure 10b shows the UV-Vis spectra for the DAP commercial fertilizer, evidencing characteristic signals of nitrogen sources, phosphate ions, and other compounds in fertilizer. Figure 10c shows the UV-Vis spectra of the biopolymer, where starch and DAP signals are present, and it includes electronic transitions of CNSL chemical compounds. Notably, the absorbance of the synthesized biopolymer decreases about the absorbance of starch and DAP, as a product of the mixture of the biopolymer components in its matrix. Therefore, the spectrum is the sum of the signals of all compounds involved in the biopolymer.

**Figure 11:**
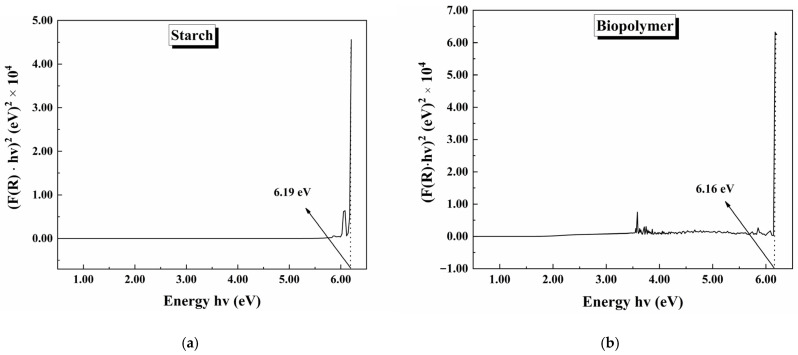
Kubelka–Munk plot for (**a**) starch and (**b**) starch–CNSL–DAP biopolymer.

Figure 11 shows Kubelka–Munk (K-M) plots using reflectance data (%R) and plotting (F(R)·hv)^2^ on the y-axis and hv on the x-axis. The K-M plot is important for calculating bandgap energy with the following expression: (F(R)·hv)^2^ = A(hv − E_g_), where the mathematical expression F(R) = (1-R)^2^/2R defines the reflectance K-M function; E_g_ is the bandgap energy; and hv is the energy. The E_g_ bandgap energy is obtained when the K-M function equals zero [41,42]. The K-M plot for starch is shown in Figure 11a, where the bandgap energy is 6.19 eV. In Figure 11b, the K-M plot for the biopolymer is shown. The bandgap energy was 6.16 eV. There was no significant difference in bandgap energy between starch and the starch–CNSL–DAP biopolymer. These materials act as insulators due to their great bandgap energy values.

### 3.5. Thermal Analysis

**Figure 12:**
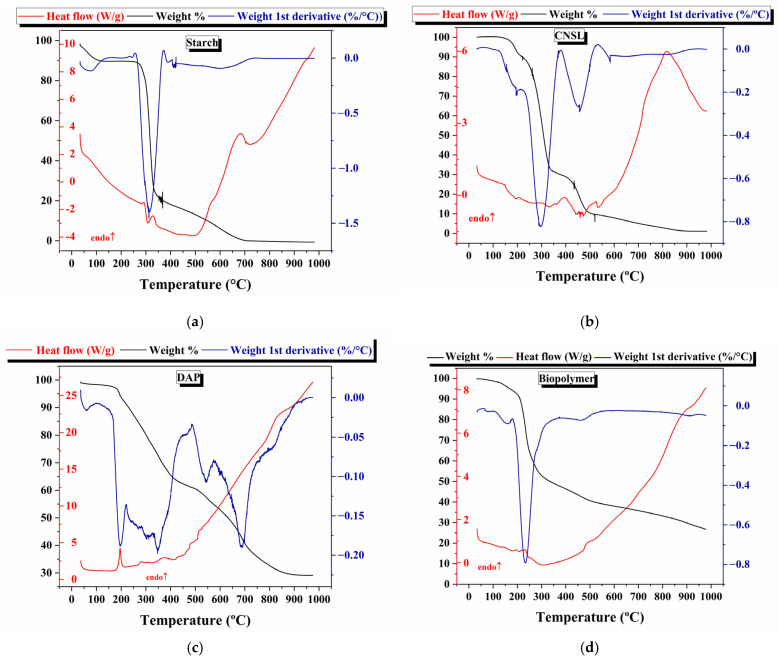
Differential scanning calorimetry (red), thermogravimetric analysis (black), and differential thermic analysis (blue) analysis for (**a**) starch, (**b**) CNSL, (**c**) DAP, and (**d**) biopolymer synthesized.

Figure 12 shows the DSC and TGA/TGA analyses for starch, CNSL, DAP, and the biopolymer. The starch thermal analysis is shown in Figure 12a. At 100 °C, 10% weight loss associated with water in starch occurs. This process is endothermic. A broad signal at 300 °C is attributed to starch degradation, where approximately 70% of weight loss occurs. After 400 °C, 20% of the weight attributed to remanent organic compounds is oxidized [43]. Similar results were reported by Pigłowska and coworkers [44]. Figure 12b shows the CNSL thermal analysis. The signal at 300 °C is associated with the degradation of CNSL compounds with a lower molecular mass, and, at high temperatures, the oxidation of organic compounds remaining in the sample occurs. Abrego and coworkers [45] found similar signals associated with anacardic acid conversion to cardanol (179 °C). Figure 12c shows the thermal behavior of DAP fertilizer. At 197 °C, diammonium phosphate reacts to eliminate ammonia (NH_3_), and H_3_PO_4_ formation occurs. At 350 °C, phosphoric acid converts into H_4_P_2_O_7_, and this compound dehydrates, forming P_4_O_10_ at temperatures higher than 580 °C, and P_4_O_10_ oxide sublimates [35].

Figure 12d shows the starch–CNSL–DAP biopolymer. The thermal process for all the compound signals overlaps in the biopolymer, mainly the signals at 100 °C (water elimination) and 250 °C (starch degradation). DAP signals in the biopolymer are not present due to the composition of the synthesized polymers. It evidences an exothermic process due to the degradation of organic compounds, both starch and CNSL compounds. The oxidation process occurs at higher temperatures as the formation of carbon dioxide and water.

## 4. Conclusions

A starch, CNSL, and DAP commercial fertilizer biopolymer composite was synthesized. Starch and CNSL were extracted from natural sources, and DAP fertilizer was used commercially. SEM analysis showed that the polymeric matrix covered the DAP fertilizer particles with starch and CNSL. Biopolymer chemical analysis revealed that C, O, P, Na, and F’s elemental composition was consistent with the synthesized polymeric matrix. FTIR analysis found vibrational modes associated with starch, CNSL, and DAP fertilizer in the synthesized biopolymer. The crystalline structure of every component in the biopolymer was evaluated by X-ray diffraction. Starch showed amorphous (21.96%) and crystalline (78.04%) phases. A- and B-type amylose crystalline structures were evidenced. The DAP fertilizer showed a crystalline structure of diammonium phosphate. Biopolymer crystallinity decreased when compared with starch. Diffraction peaks in biopolymer overlapped signals between starch and DAP fertilizer. The electronic transitions of the materials were studied using UV-Vis spectroscopy. Electronic transitions associated with starch, CNSL, and DAP were registered in the biopolymer, showing possible signal broadenings/overlappings. By reflectance measurements, bandgap calculations of starch and the starch–CNSL–DAP biopolymers were found through the Kubelka–Munk equation. Thermal behavior was studied using DSC/TGA. Water elimination, starch degradation, oxidation, and gas formation were registered for the starch–CNSL–DAP biopolymer.

## Figures and Tables

**Table 1:** Semiquantitative chemical analysis measurements of the synthesized biopolymer.

Element	Atomic %	Atomic % Error
C	38.5	0.2
O	52.0	0.4
F	4.0	0.3
Na	1.0	0.1
P	3.4	0.1
K	1.1	0.1

## Data Availability

The original contributions presented in this study are included in this article, further inquiries can be directed to the corresponding author.
